# Updating the Phylogenetic Dating of New Caledonian Biodiversity with a Meta-analysis of the Available Evidence

**DOI:** 10.1038/s41598-017-02964-x

**Published:** 2017-06-16

**Authors:** Romain Nattier, Roseli Pellens, Tony Robillard, Hervé Jourdan, Frédéric Legendre, Maram Caesar, André Nel, Philippe Grandcolas

**Affiliations:** 1 Institut de Systématique, Evolution, Biodiversité, ISYEB - UMR 7205 CNRS MNHN UPMC EPHE, Muséum national d’Histoire naturelle, Sorbonne Universités, CP 50, 57 rue Cuvier, 75005 Paris, France; 2 Institut Méditerranéen de Biodiversité et d’Ecologie marine et continentale (IMBE), Aix Marseille Univ., Univ. Avignon, CNRS, IRD, Centre IRD Nouméa, BP A5, 98848 Nouméa Cedex, New Caledonia

## Abstract

For a long time, New Caledonia was considered a continental island, a fragment of Gondwana harbouring old clades that originated by vicariance and so were thought to be locally ancient. Recent molecular phylogenetic studies dating diversification and geological data indicating important events of submergence during the Paleocene and Eocene (until 37 Ma) brought evidence to dismiss this old hypothesis. In spite of this, some authors still insist on the idea of a local permanence of a Gondwanan biota, justifying this assumption through a complex scenario of survival by hopping to and from nearby and now-vanished islands. Based on a comprehensive review of the literature, we found 40 studies dating regional clades of diverse organisms and we used them to test the hypothesis that New Caledonian and inclusive Pacific island clades are older than 37 Ma. The results of this meta-analysis provide strong evidence for refuting the hypothesis of a Gondwanan refuge with a biota that originated by vicariance. Only a few inclusive Pacific clades (6 out of 40) were older than the oldest existing island. We suggest that these clades could have extinct members either on vanished islands or nearby continents, emphasizing the role of dispersal and extinction in shaping the present-day biota.

## Introduction

Island biology has spurred scientific innovation and has been crucial to the formulation of new paradigms since the early days of evolutionary biology^1–6^. More recently, it stimulated a methodological discussion on molecular phylogenetic dating and the nature of biogeographical events^7–9^. Central to this discussion is the case of large Gondwanan islands, such as Madagascar^10^, and some islands that are traditionally considered to be continental, but which are probably oceanic, such as New Zealand^11^ and New Caledonia.

The biogeographical paradigm for the origin of the New Caledonian biota has been profoundly revisited over the last ten years e.g. refs 12–15. The traditional perspective considers the main island Grande Terre (hereafter NC) to be a small piece of the Gondwanan continent that separated from Australia 80 million years ago. This view mainly relies on NC’s old geological basement and the local occurrence of relicts^16–19^. In fact, as summarized in recent reviews^13, 20^, geological studies have concluded that the island has a very complex sedimentary and tectonic history with several long episodes of submergence and a final emergence dated at 37 (±3) Ma^21–26^. Despite the progressive accumulation of independent phylogenetic evidence for post-emergence colonization (reviewed in refs 13, 27 and 28), some authors are still reticent to adopt this new view, as epitomized by M. Heads^7^. This author argued that i) molecular dating could be corrupted by poor calibrations and ii) lineages could be considered to be locally permanent if they hopped between neighbouring islands. Methodological as well as theoretical discussions in defence of molecular dating have been recently addressed^8, 9^. We will not discuss here the ways of testing dating hypotheses, which is a rich and generally well-understood subject. The second statement refers to a less commonly broached topic which needs to be clearly formulated in order to be subjected to a more rigorous hypothesis testing. Island-hopping, there and back, is not equivalent to permanence on the same piece of land. It actually implies multiple short-distance dispersal events between nearby and temporary islands or other territories that do not exist anymore. Therefore, it does not support the assumption of a vicariance relationship between an island system and regional continental masses. But it might reveal interesting scenarios involving a succession of multiple short-distance dispersals. The likelihood of such island-hopping scenarios, first formulated by biogeographers e.g. refs 2, 29 and 30, can be tested by looking at whether the age of an insular clade is older than the oldest island or seamount in an island system^15, 31^.

In relation to these two assumptions, an additional and important problem is that the origin of the NC biota is often inferred by generalizing from selected lineages in a narrative way^7, 32^, when hypothesis testing should be favoured^33, 34^.

Here, we wanted to break from the narrative arguments by compiling all available evidence concerning the diversification of local clades and their history within the Pacific insular system in order to estimate the age of the New Caledonian biota in the light of present scientific results^13, 27, 28^.

We aimed to answer two main questions: (1) How old is the New Caledonian biota? (2) Are regional insular clades older than the oldest island in the region? Based on a meta-analysis of all available phylogenetic data, we should be able to conclude for or against the Gondwanan vicariance hypothesis (i.e. the island biota older is than the geological submergence of the island), and assess the relative importance of island-hopping as a mechanism for shaping both the regional and NC biotas.

## Results

We found 76 published phylogenetic analyses with divergence-date estimates for New Caledonian clades; however only 40 of these estimated the divergence-date of both the NC crown group and the corresponding Pacific group. These studies cover diverse groups of arthropods (21 studies), plants (16), vertebrates (2), and molluscs (1) (Table 1). In 30 of these studies, the age of the NC crown group and the inclusive Pacific island group was the same because they are the same group (i.e. all species are from NC).

**Table 1:** Details of the studies included in this review: taxonomical information, divergence age estimates for NC crown group and inclusive Pacific crown group, and reference number.

#	Clade name	Taxonomic information	Crown age of the NC group (Ma)	Crown age of the inclusive Pacific group (Ma)	Ref.
1	*Apsilochorema caledonicum*	Arthropoda: Hexapoda: Trichoptera: Hydrobiosidae	0.9 (3.5–0)	0.9 (3.5–0)	62
2	*Scaevola*	Plantae: Asterales: Goodeniaceae	2	5.3	63
3	*Angustonicus*	Arthropoda: Hexapoda: Blattodea: Blattidae	2.7 (4.0–1.3)	4.7	12
4	*Clinosperma and Cyphokentia*	Plantae: Arecales: Arecaceae	3.3	3.3	64
5	*Leptomyrmex*	Arthropoda: Hexapoda: Hymenoptera: Formicidae	4.1 (6.9–1.4)	4.1 (6.9–1.4)	65
6	*Caledonula*	Arthropoda: Hexapoda: Orthoptera: Oxyinae	4.9 (7.5–2.9)	4.9 (7.5–2.9)	66
7	*Dracophyllum*	Plantae: Ericales: Ericaceae	5.2 (7.2–0.7)	5.2 (7.2–0.7)	67
8	*Dacrydium*	Plantae: Araucariales: Podocarpaceae	5.6	10 (20–5)	68
9	*Piliocalyx*	Plantae: Myrtales: Myrtaceae	6 (2.5–13.5)	6 (2.5–13.5)	69
10	*Psychotria*	Plantae: Rubiales: Rubiaceae	6.9 (9.8–4.6)	6.9 (9.8–4.6)	70
11	*Diospyros*	Plantae: Ericales: Ebenaceae	7.2 (10.1–4.5)	9.1 (6–13)	71
12	*Geissois*	Plantae: Oxalidales: Cunoniaceae	7.3 (12.8–3.5)	7.3 (12.8–3.5)	72
13	*Grammitis*	Plantae: Polypodiales: Polypodiaceae	8.5 (12.8–5)	10 (13.8–5.8)	73
14	*Picrella*	Plantae: Sapindales: Rutaceae	8.6 (11.7–5)	8.6 (11.7–5)	74
15	*Pauropsalta johanae* and *Myersalna depicta*	Arthropoda: Hexapoda: Hemiptera: Cicadidae	8.9	9.7	75
16	*Lordomyrma*	Arthropoda: Hexapoda: Hymenoptera: Formicidae	9	9	76
17	*Pheidole*	Arthropoda: Hexapoda: Hymenoptera: Formicidae	9	9	77
18	*Rhantus*	Arthropoda: Hexapoda: Coleoptera: Dytiscidae	9	9	78
19	*Bavayia sauvagei* and *Rhacodactylus leachianus*	Vertebrata: Squamata: Diplodactylidae	9.6	9.6	79
20	*Necterosoma*	Arthropoda: Hexapoda: Coleoptera: Dytiscidae	10	10	80
21	*Agnotecous*	Arthropoda: Hexapoda: Orthoptera: Gryllidae	10.3 (17.9–4.9)	15.3 (21.4–9.7)	15
22	*Papuadytes*	Arthropoda: Hexapoda: Coleoptera: Dytiscidae	10.9	10.9	81
23	*Cryptops pictus*	Arthropoda: Chilopoda: Cryptopidae	11.7 (23.7–2.9)	11.7 (23.7–2.9)	49
24	*Arsipoda*	Arthropoda: Hexapoda: Coleoptera: Chrysomelidae	12.8 (24.1–6.5)	12.8 (24.1–6.5)	82
25	*Zygonum*	Plantae: Magnoliales: Winteraceae	15 (25–5)	15 (25–5)	83
26	*Nothofagus*	Plantae: Fagales: Nothofagaceae	16.4 (27.5–6.7)	16.4 (27.5–6.7)	84
27	*Planchonella*	Plantae: Ebenales: Sapotaceae	17.4 (12.3–23.5)	17.4 (12.3–23.5)	31
28	*Phyllanthus*	Plantae: Malpighiales: Phyllanthaceae	20 (27.2–17.7)	20 (27.2–17.7)	85
29	Gekkota	Vertebrata: Squamata: Diplodactylidae	22.4 (18.4–26.5)	22.4 (18.4–26.5)	86
30	*Agmina*	Arthropoda: Hexapoda: Trichoptera: Ecnomidae	22.5 (25.7–17.7)	22.5 (25.7–17.7)	87
31	*Pycnandra*	Plantae: Ericales: Sapotaceae	22.9 (29–17.6)	22.9 (29–17.6)	88
32	*Hemistomia*, *Kanakyella* and *Leiorhagium*	Mollusca: Littorinimorpha: Tateidae	24.6 (15.8–34.1)	—	89
33	*Delarbrea* and *Myodocarpus*	Plantae: Apiales: Myodocarpaceae	25.4 (7.9–47.3)	25.4 (7.9–47.3)	90
34	*Orthopsyche* and *Caledopsyche*	Arthropoda: Hexapoda: Trichoptera: Hydropsychidae	28.2 (32.5–22.4)	30.6	91
35	*Dolichoris*	Arthropoda: Hexapoda: Hymenoptera: Agaonidae	40.3 (64.5–20.7)	40.3 (64.5–20.7)	27
36	*Lanceocercata*	Arthropoda: Hexapoda: Phasmoptera: Phasmatidae	41.1 (55.4–29.1)	41.1 (55.4–29.1)	92
37	*Zalmoxis*	Arthropoda: Arachnida: Opiliones: Zalmoxidae	48.9 (65.8–37.1)	72.6	50
38	*Sabatinca*	Arthropoda: Hexapoda: Lepidoptera: Micropterigidae	52 (64–40)	82.1 (107.4–66.3)	52
39	Eumolpinae	Arthropoda: Hexapoda: Coleoptera: Chrysomelidae	59.9 (71–50.1)	59.9 (71–50.1)	51
40	*Troglosiro*	Arthropoda: Arachnida: Opiliones: Troglosironidae	57 (73–40)	57 (73–40)	93

### Gondwanan Vicariance or Refuge Hypothesis

**Figure 1:**
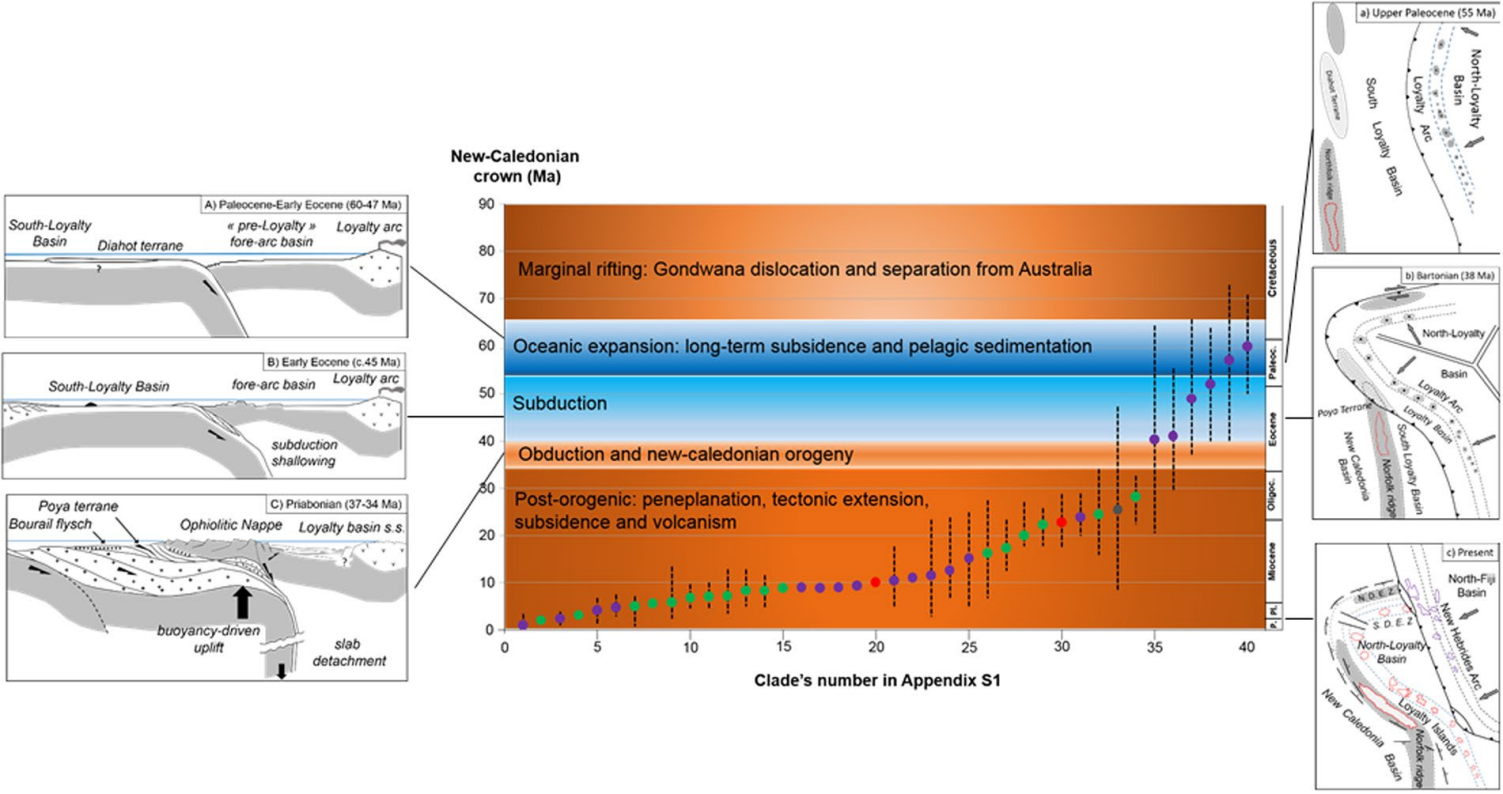
Estimated crown age of New Caledonian clades from 40 selected studies (purple: arthropods, green: plants, red: vertebrates, grey: molluscs). Dotted lines indicate confidence intervals when available. A–C: tectonic/geodynamic model of the evolution of the Eocene accretion/subduction complex of New Caledonia; (a–c): reconstruction of the convergence of the Norfolk Ridge and the Loyalty Arc. A-C and a-c are not to scale and redrawn from^23^.

**Figure 2:**
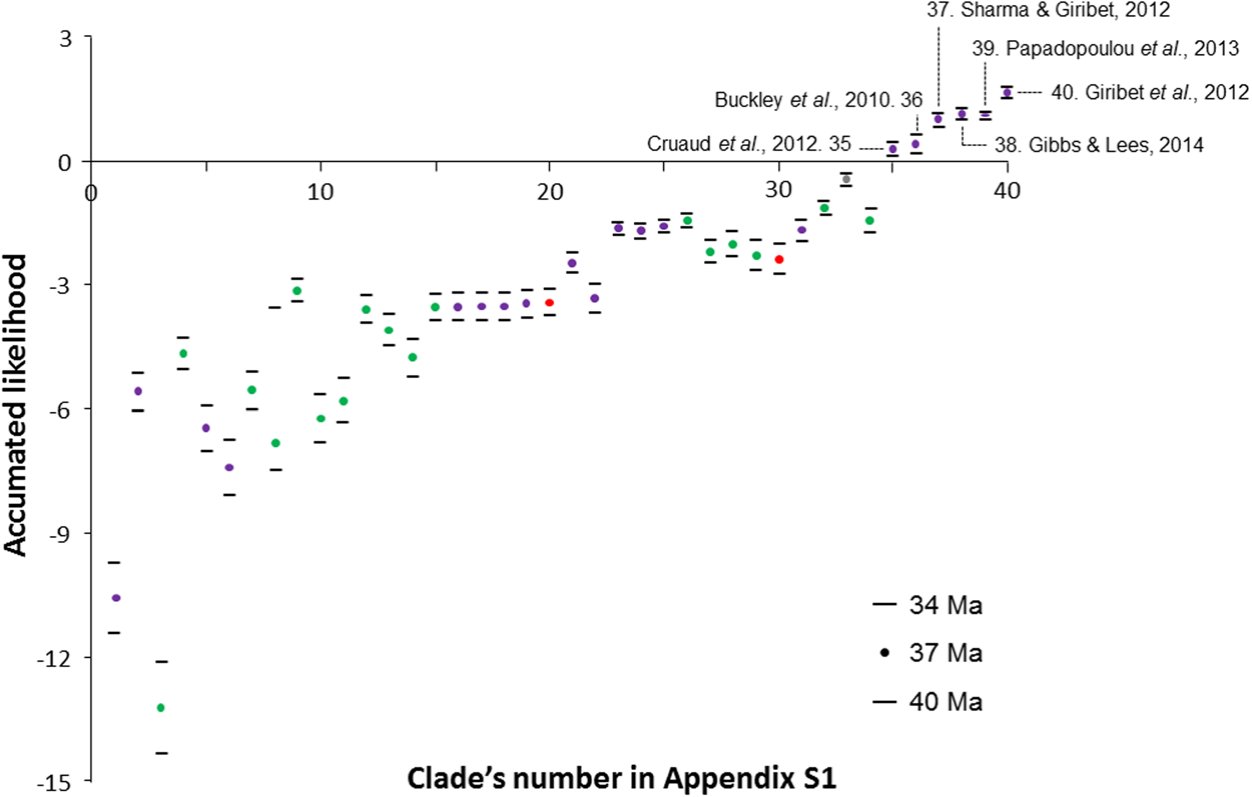
Accumulated likelihood of being older than 37 Ma (±3 Ma). Numbers on the X-axis refer to the Clade ID in Table 1. Purple: arthropods, green: plants, red: vertebrates, grey: molluscs.

Taking confidence intervals into account, in 33 phylogenies (82.5%) clades (crown age) were estimated to be strictly younger than the emergence of NC, in six studies (15%) the crown age predated 37 ± 3 Ma, and two studies presented a clade that overlapped this period (Fig. 1). The probability values for most of these groups were either negative or around zero (mean = −2.82; median = −2.93; SE = 0.44, n = 40). For the six groups estimated to be older than 37 Ma, the probability values were positive but still very low (i.e. lower than 2.00), indicating a very small departure from the 37 ± 3 Ma range. This shows that being younger than the emergence of NC is a feature that is common to the great majority of NC groups (Fig. 2).

### Island-Hopping Hypothesis

**Figure 3:**
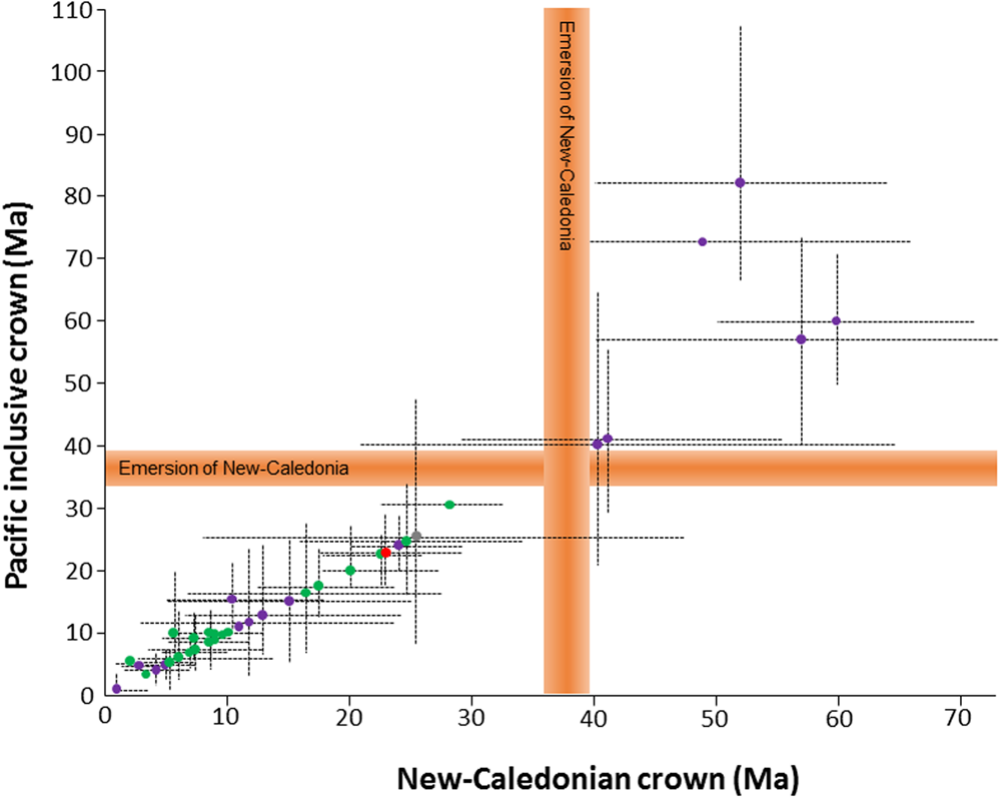
Estimated crown age of the New Caledonian and Pacific island clades from the 40 selected studies. Dotted lines indicate confidence intervals when available. Purple: arthropods, green: plants, red: vertebrates, grey: molluscs.

**Figure 4:**
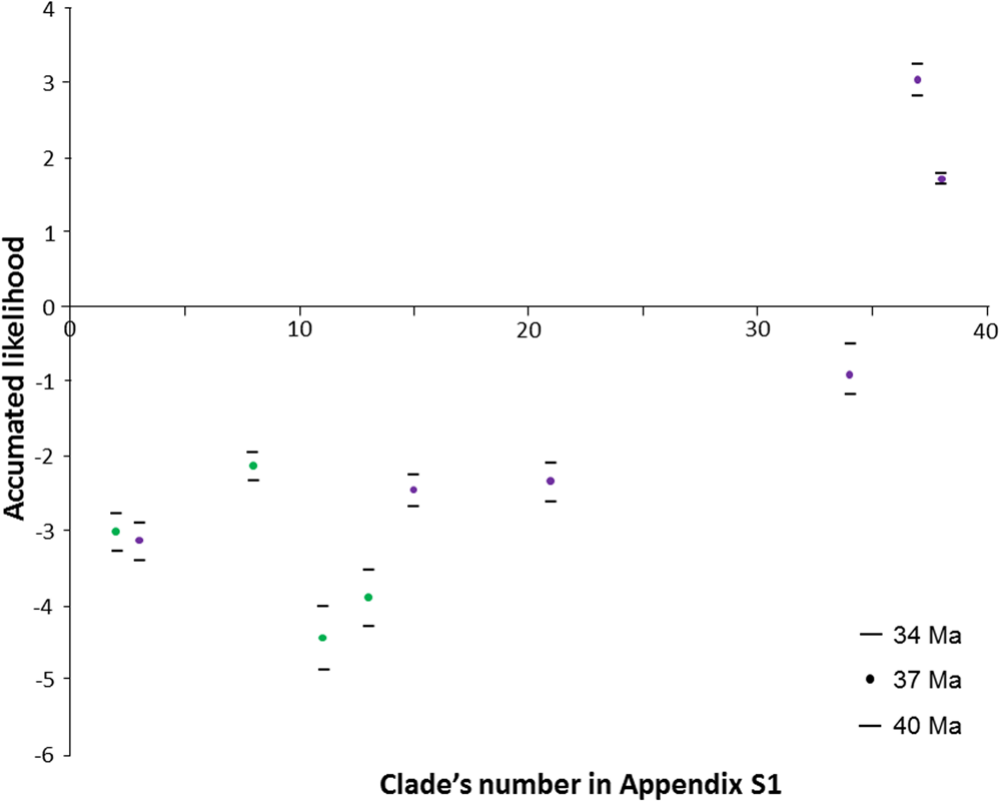
Accumulated likelihood of the inclusive Pacific Group being older than 37 Ma (±3 Ma), from studies where crown age and Pacific insular age differ (n = 10). Numbers on the X-axis refer to the Clade ID in Table 1. Purple: arthropods, green: plants, red: vertebrates, grey: molluscs.

As shown above, only six studies (all on arthropods) estimated that the New Caledonian and Pacific inclusive clades were older than 37 Ma (Figs 3 and 4). This means that there are few old groups in the region, contrary to the expectations of the Gondwanan refuge hypothesis. Taking the dataset as a whole, the probabilities of the NC crown group and the inclusive Pacific group being older than 37 Ma are highly correlated (rs = 0.931022, p < 0.0001). Even if the inclusive Pacific crown groups are logically often older than NC crown groups that they include (Wilcoxon V = 455; p < 0.001), their probability values still tend to be negative (Mean values = −1.48; Median = −0.56; SE = 0.63, n = 15). This provides evidence that most of them are much younger than 37 Ma.

## Discussion

After several decades of intense research, we now have a very significant number of molecular phylogenetic and dating studies that provide new insights regarding the diversification of the biota of the Pacific region. Our meta-analysis has allowed us to test the most classical hypotheses regarding the origins of the biota of New Caledonia. Could the New Caledonian biota be traced back directly to Gondwanan times and its geography (the so-called Gondwanan refuge/vicariance hypothesis)? Did local groups evolve before the emergence of the oldest present-day islands by hopping between now-submerged islands?

Our analysis supports previous conclusions based on age estimates of a single or a few taxonomic groups^13, 27, 28^, but also allows us to draw more general conclusions and perspectives as it takes into account a much larger array of taxonomic groups (arthropods, molluscs, vertebrates, plants). First of all, the Gondwanan refuge hypothesis can no longer be considered a major possibility for explaining the origin of the NC biota. The New Caledonian origin of thirty-six out of forty groups studied does not predate the emergence of NC in the Eocene. The four remaining groups are not much older than the island itself (between 37.1 and 50.1 Ma) and therefore do not refer to a time that is anterior to the breakup of Gondwana (80 Ma at the very least^24^). It means that, for most groups, local diversification mainly resulted from colonization by long- or short-distance dispersal, generally long after the island’s main emergence 37 Ma.

Given that molecular trees were calibrated without reference to the emergence of New Caledonia, this statement is independent from and consistent with many detailed geological studies (recently reviewed in refs 24 and 26). Even if the Gondwanan refuge hypothesis cannot be considered to be a major explanation, New Caledonia and the local groups of organisms that have been studied are not particularly young. Compared to New Zealand or Madagascar, New Caledonia is not a large island (about 16,000 km^2^). Nonetheless, it is actually a very old oceanic island, probably the oldest in the world (37 Ma as a minimum), and most local diversifications are remarkably ancient (half of them originated between 10 and 35 Ma). Inferring the age of local biomes (i.e. forests versus maquis), however, is quite difficult from this dataset because the oldest groups often inhabited different vegetation types and the internal processes of diversification and habitat change can be complex^35, 36^. As a matter of comparison, the origins of other island biodiversity hotspots with a Gondwanan substrate have been recently reviewed, with similar conclusions: in Madagascar, most of the present-day biota is of Cenozoic origin, with overseas dispersal from Africa favoured by oceanic paleocurrents^10, 37, 38^; and similarly for the New-Zealand biota^39, 40^, which derives from long-distance dispersal.

The second classical hypothesis regarding the origin of the New Caledonian biota suggests that some organisms hopped between now-submerged islands. It has often been assumed that old groups of organisms could have remained locally present in this way^7, 29, 36^, an assumption that can be tested by looking at whether the age of an insular clade is older than that of the oldest island^15, 31^.

Some recent geological studies have assessed the regional presence of now-submerged islands that were emerged during the period when New Zealand and New Caledonia were submerged^18, 21, 23, 41–45^. Even though their precise occurrence near NC at the right time to allow island hopping has been proposed^46–48^ but not proven yet, the argument deserves proper consideration^12, 13, 15^.

Our meta-analysis collected evidence regarding the age of inclusive Pacific island clades and showed that four interesting clades are estimated to be older than 37 Ma. Two of them are opilionids, an old group whose age estimate is stretched by some very early fossil arthropods^49, 50^. Another group (beetles)^51^ has a very wide age estimate range (between 24.10 and 70,97 Ma for the New Caledonian clade), and the last group’s (jaw-moths) youngest age (40 Ma)^52^ is estimated to be close to the emergence geological date of NC. It could be inferred that these lineages may have occurred in some terrestrial areas long ago where they went extinct and survived by hopping to New Caledonia^13, 15^. The details of this island hopping scenario remain to be established. In this respect, certain assumptions regarding short-distance dispersal (SDD, from islands) have been arbitrarily favoured by many authors^2, 30, 46, 53^, probably as result of a preference over the assumptions regarding long-distance dispersal (LDD, from continents). SDD is probably seen as more compatible with the biological characteristics of certain organisms^48^ even though it generally involves a more complex and speculative combination of low probability events^33^ than a single LDD event. However, focusing solely on islands^46^ could be misleading since it precludes testing certain hypotheses by arbitrarily excluding LDD events and adjacent continental areas such as Australia or New Zealand from the scope of study. Rather than assuming a speculative preference for a special dispersal mechanism, we invite scientists to test these different possibilities.

According to molecular dating, a small number of groups, all belonging to arthropods (opilionids, beetles, jaw-moths) evolved by hopping/local dispersal mechanisms between terrestrial lands in the region. As shown by the strong correlation between the age of the NC and Pacific clades, a group that diversified long ago in the Pacific region has been more prone to colonize and to diversify earlier in New Caledonia. In the future, it will be interesting to verify whether the trend that arthropods belong to old lineages is confirmed. At the present time, given the ecological diversity and the size of the arthropod groups already studied, no clear conclusions can be drawn concerning their putative dispersal abilities.

The trends highlighted here can guide future research. First of all, some groups are regionally too young to allow us to test different hypotheses within the full-time window of regional paleogeography from the Gondwana breakup to the emergence of New Caledonia at the end of Eocene. Therefore, there should be a focus on studying either old or poorly known groups that could be found to be older than previously thought when more extensively sampled. In the recent case of an insect group e.g. refs 15 and 35, a dedicated field-sampling program has shown that the putative age of the lineage was much older than what was previously known, and detected an interesting case of relictness^54^. Another issue would be to study in more detail monotypic New Caledonian relicts in order to distinguish crown from stem groups^34^. For example, *Amborella trichopoda* is a remarkable relict whose phylogenetic position places it alone on a long branch apparently sister to all flowering plants, but whose biogeographic significance remains paradoxically difficult to interpret^34, 55^. Understanding the evolution of biota from past occurrences of taxa found in the fossil record should also shed light on this subject and should be considered a priority at the time being. The fossil collection of New Caledonia, which is presently very poor, could easily be improved^56^ as the island has an amazing geological diversity^57^. An augmented fossil record would considerably improve our understanding of the evolution of this biota in terms of its distribution and extinction, and serve as fruitful confrontation and integration points when combined with molecular phylogenies.

## Material and Methods

### Hypotheses testing

The Gondwanan Vicariance Hypothesis. This hypothesis states that the biota of New Caledonia was formed by vicariance from the Gondwana continent around 80 Ma and that archaic groups have survived and diversified through local and long-term cladogenesis. If this hypothesis is correct, we would expect a majority of New Caledonian clades to be older than 37 Ma, the date of emergence of the island.

The Island-Hopping Hypothesis. This hypothesis states that New Caledonian taxa were part of a regional pool and have survived by hopping between various now-submerged islands at a time when New Caledonia was submerged. In this case, we would expect the inclusive Pacific island clade to be older than the date of emergence of the oldest Pacific island, which is in fact the date of emergence of New Caledonia i.e. 37 ± 3 Ma.

### Data Collection

We scoured the literature in search of molecular phylogenies containing New Caledonian taxa and divergence-date estimates. Queries were made in Google Scholar and Web of Sciences using the following keywords: New Caledonia, molecular dating, phylogeny, biogeography (up to the 15^th^ February 2016). We found studies focusing entirely on New Caledonian clades as well as studies on regional or much wider geographic scales. When several studies dated the same New Caledonian group, we considered only the most recent or most complete publication (i.e. the one with the largest taxonomic sampling of New Caledonian species). In studies with several date estimates (obtained for example using different methods or calibration schemes), we considered only the oldest estimate, in order to be more conservative in our tests.

Two different ages were recorded from each study: the age of the New Caledonian group and the age of the inclusive Pacific island group. The latter was defined by considering oceanic islands in the whole Pacific region (including Hawaiian islands). We based our analysis on the ages of crown groups because this provides direct information regarding the time and place of the groups’ diversification^33, 58–60^.

For each study, we recorded the confidence interval of age estimates and reported the method and calibration scheme (Table 1). We were careful to only select studies where estimates were calculated independently from the local geological history, i.e. excluding studies in which the emergence of NC was used as a calibration point.

### Statistical Analyses

We used an accumulated likelihood method^61^ to assess whether New Caledonian clades and inclusive Pacific island groups are older than 37 Ma. Accumulated likelihood values are a function of the time difference between the age of the clade and the emergence date of NC. This method assigns higher scores to clades that are much older than the NC emergence, lower scores to taxa with divergence times close to 37 Ma, and negative scores to much younger taxa. These scores were calculated by determining the distance of the confidence intervals from the 37 Ma emergence date, and then dividing these values by the total confidence interval (reflecting the uncertainty in the phylogenetic time frame). This calculation estimates the number of times the node age range coincides with the emergence date of NC. Since these data can be partitioned into three major groups, i.e. clades that are younger, older or intersecting the 37 Ma time frame, we used three equations to assess the probability:

1 $${P}_{c(p)}=(Mx-E)/(Mx-Mn)$$

2 $${P}_{c(n)}=(Mn-E)/(Mx-Mn)$$

3 $${P}_{c(i)}=\{[Mn+(Mx-Mn)/2]-E\}/[(Mx-Mn)/2]$$

where *Mx* is the maximum divergence time (oldest date), *Mn* is the minimum divergence time (youngest date) and E is the emergence date (here fixed at 37 Ma). As *per* Cody *et al. (*2010), in order to include clades without previously calculated confidence intervals or for which the maximum and minimum divergence times were identical (i.e. Mx-Mn = 0), we used a default range value equal to the 10^th^ percentile of all differences between Mx and Mn, i.e. of 4.6 Ma for the New Caledonian crown group and of 7.0 Ma for the Pacific crown group.

We used eq. 1 (P_c(p)_) when the minimum value of the confidence interval was greater than 37 Ma; eq. 2 (*P*_*c(n)*_) when the maximum value of the confidence interval was less than 37 Ma; and eq. 3 (*P*_*c(i)*_) when confidence intervals intersected the emergence date, i.e. where the maximum was greater than 37 Ma and the minimum smaller than 37 Ma. Scores greater than zero indicate the probability that the clade is older than the emergence of NC and scores below zero indicate the probability of being younger than the emergence of NC. The greater the positive score, the earlier the minimum divergence date occurred before 37 Ma. The greater the negative score, the later the maximum divergence date occurred after 37 Ma.

In order to test whether the probability (here represented by the score values) of being older than emergence of NC is related to the age of the Pacific groups, we used a Mann-Whitney U test and a Spearman correlation test.
